# Range expansion of the Asian native giant resin bee *Megachile sculpturalis* (Hymenoptera, Apoidea, Megachilidae) in France

**DOI:** 10.1002/ece3.3758

**Published:** 2018-01-02

**Authors:** Violette Le Féon, Matthieu Aubert, David Genoud, Valérie Andrieu‐Ponel, Paul Westrich, Benoît Geslin

**Affiliations:** ^1^ Observatoire des Abeilles Arzens France; ^2^ CNRS, IRD, IMBE Aix‐Marseille University University of Avignon Marseille France; ^3^ Institut für Biologie und Naturschutz Kusterdingen Germany

**Keywords:** bees, cavity nesters, citizen‐reported data, competition, introduced species, plant–pollinator interactions, range expansion dynamics

## Abstract

In 2008, a new species for the French bee fauna was recorded in Allauch near Marseille: the giant resin bee, *Megachile sculpturalis* (Smith, 1853). This was the first European record of this species that is native to East Asia. To our knowledge, it is the first introduced bee species in Europe. Here, we provide an overview of the current distribution of *M. sculpturalis* in France and we describe the history of its range expansion. Besides our own observations, information was compiled from literature and Internet websites, and by contacting naturalist networks. We collected a total of 117 records (*locality* × *year* combinations) for the 2008–2016 period. The geographical range of *M. sculpturalis* has extended remarkably, now occupying a third of continental France, with the most northern and western records located 335 and 520 km from Allauch, respectively. Information on its phenology, feeding, and nesting behavior is also provided. We report several events of nest occupation or eviction of *Osmia* sp. and *Xylocopa* sp. individuals by *M. sculpturalis*. Our results show that *M. sculpturalis* is now well established in France. Given its capacity to adapt and rapidly expand its range, we recommend amplifying the monitoring of this species to better anticipate the changes in its geographical range and its potential impacts on native bees.

## 1 INTRODUCTION

The giant resin bee *Megachile* (*Callomegachile*) *sculpturalis* (Smith, 1853) is a large species native to East Asia (Japan, China, and Korean peninsula; Hinojosa‐Díaz, 2008; Wu, 2005). In 1994, *M. sculpturalis* was observed for the first time outside its native geographical range in North Carolina, USA (Mangum & Brooks, 1997). Since this first observation, the species has rapidly expanded throughout eastern USA (Mangum & Sumner, 2003; Parys, Tripodi, & Sampson, 2015) and reached Canada (Ontario) in 2002 (Paiero & Buck, 2003). It continued its westward expansion and currently occurs in Texas and Kansas (Hinojosa‐Díaz, 2008; Parys et al., 2015).

The important capacity of adaptation and the fast expansion of *M. sculpturalis* are partly explained by its generalist (polylectic) diet, its good flight ability, and its nesting behavior (Parys et al., 2015). Indeed, *M. sculpturalis* is a cavity‐nester that uses holes in wood and stems and thus its introductions could result from transportation in wood or other nesting substrates. Moreover, the species has been reported using human‐made nesting structures (“bee hotels”) (Fortel, Henry, Guilbaud, Mouret, & Vaissière, 2016; Quaranta, Sommaruga, Balzarini, & Felicioli, 2014). The increasing use of these structures as a conservation tool for bees (MacIvor & Packer, 2015) could have promoted its spread.

Due to their crucial role as pollinators, bees are perceived to be beneficial and, comparatively to other biological groups, few concerns have been paid to the introduced bees. Nevertheless, in Oceania and North and South America, many examples of bee introductions and their subsequent deleterious consequences for native bee and plant species have been described in the literature (Geslin et al., 2017; Goulson, 2003). Introduced bees could either compete directly for nesting or feeding resources or indirectly by modifying the whole plant–pollinator network through enhancing the reproduction of exotic plant species (e.g., *Lupinus arboreus* in Tasmania subsequently to the invasion of *Bombus terrestris*, Stout, Kells, & Goulson, 2002).

Regarding *M. sculpturalis*, competition for nesting resources and aggressive behaviors toward other bee species have been previously reported. Indeed, in the United States, Laport and Minckley (2012) and Roulston and Malfi (2012) described aggressive evictions and occupations of nests of a native bee species (*Xylocopa virginica* L.) by *M. sculpturalis*.

In contrast with North and South America, where many introduced bees have been detected, bee introductions have been rare in European countries. To our knowledge (Goulson, 2003; Russo, 2016), *M. sculpturalis* is the first introduced bee species in Europe, where it has been detected for the first time in 2008. The observation occurred in Allauch near Marseille, France (Vereecken & Barbier, 2009). Then, the species was reported in Italy in 2009 (Quaranta et al., 2014), in Switzerland in 2012 (Amiet, 2012), in Germany in 2015 (Westrich, Knapp, & Berney, 2015), and in Austria in 2017 (P. Westrich, unpublished data 2017).

Since the first observation in 2008, no review of the range expansion of *M. sculpturalis* in France has been reported. Here, we gathered all available data on the presence of *M. sculpturalis* in France. We documented its nesting and feeding behaviors, and the interactions with French native bees. We discuss avenues and potential consequences of this invasion and present monitoring measures.

## 2 METHODS

### 2.1 *Megachile sculpturalis*

**Figure 1:**
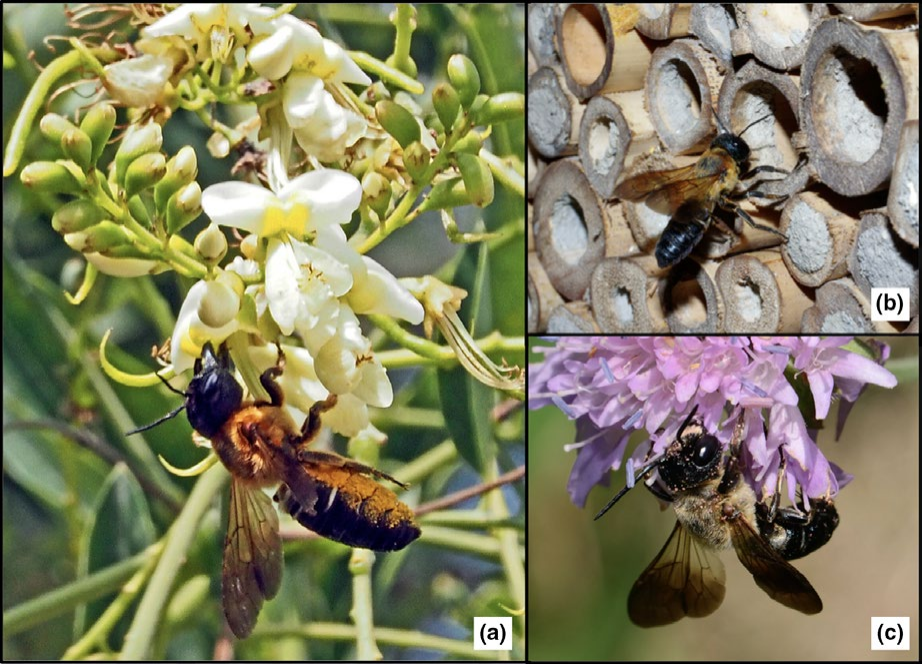
Photographs of *Megachile sculpturalis* taken in France: (a) a female on *Sophora japonica* (at Bouillargues in 2014 © Danièle Tixier‐Inrep); (b) a female at the entrance of an *Arundo* sp. stem (at Avignon in 2015 © Daniel Mathieu); (c) a male drinking nectar on *Scabiosa columbaria* (at Matemale in 2013 © David Genoud)

Bee identification at the species level usually requires a high level of taxonomic expertise and is mostly impossible without collecting specimens and identifying them using both relevant literature and large reference collections. However, in our studied area, *M. sculpturalis* can be easily differentiated from other bee species: it is one of the largest bee species in France, with a size range from 22 to 27 mm long for females and from 14 to 19 mm long for males (Paiero & Buck, 2003), it has infuscated wings, and its thorax is covered with orange hair (Figure 1). Therefore, this species is easily recognizable for an informed naturalist in the field and in pictures.

### 2.2 Data gathering

In order to assess the current distribution as well as the history of the range expansion of *M. sculpturalis* in France, we looked for all available occurrence data. Our study covered the 2008–2016 period, and we gathered data until 31 October 2016.

First of all, we gathered our own observations and observations reported in publications (i.e., Andrieu‐Ponel et al., 2016; Fortel et al., 2016; Gihr & Westrich, 2013; Vereecken & Barbier, 2009). Secondly, we contacted entomologists interested in bees. To achieve this goal, we directly sent emails to our naturalist networks and we used the Internet discussion group (or “forum”) called “Apoidea‐Gallica” (https://fr.groups.yahoo.com/neo/groups/apoidea-gallica/info). This forum gathers French‐speaking people interested in bees, including expert taxonomists, environmental managers, and researchers. Its counts 383 participants (up to date on 16 September 2017), which are globally located in all French regions as well as in neighboring countries. On 15 February 2016, we sent a message on this forum informing about our study on *M. sculpturalis* distribution and asking for observation records. Thirdly, we looked for information on Internet websites. In total, observations were found in seven websites but the most rewarding ones were “Le Monde des insectes” (https://www.insecte.org/) and the website of the “Photographic Survey of Flower Visitors” (hereafter Spipoll, see http://www.spipoll.org/). “Le Monde des insectes” is a French‐speaking website dedicated to entomology, where expert or beginner entomologists post photographs and taxonomist experts help them to identify the photographed insect at the finest taxonomic level as possible. The Spipoll is a national monitoring program of insect pollinators based on citizen science launched in 2010 (Deguines, Julliard, de Flores, & Fontaine, 2012, 2016) based on the compilation of photographic collections of insects interacting with a plant species at a given place and time. Basically, wherever in mainland France, volunteers are asked to choose a flowering plant species and to photograph all insects either feeding or landing on the flowers over a standardized time period. The photographic collections cover the whole France (except Corsica) and contain nearly 218,000 insect photographs taken by about 1,300 volunteers in more than 2,600 different cities (up to date on May 11th, 2016; see http://rapport-spipoll-2015.semi-k.net/). Fourthly, we informed about our study the teachers from agricultural high schools involved in standardized surveys of bees in the framework of a research program presented in Le Féon et al. (2016) as well as environmental managers from the “Réserves Naturelles de France” network (natural protected areas). By doing so, we informed people located in all parts of France, which could help us to collect data.

All records were carefully verified prior to inclusion in our database. The data were validated only if a picture or a specimen was available. When the species was detected, the following questions were asked: (1) What are the location and the date of the observation? (2) How many individuals did you see? (3) Could you give the sex of individuals? (4) Were individuals foraging or nesting? (5) If applicable, what was the flowering plant species visited? or (6) the nest material used? When *M. sculpturalis* was observed at different places in the same city, we considered these observations as distinct records if the places (hereafter localities) were located at least 500 m away from each other.

## 3 RESULTS

We collected 117 records, that is, *locality* × *year* combinations, from 70 different observers. The two main sources of data were the “Apoidea‐Gallica” forum and naturalists we directly contacted (respectively, 28.2% and 19.6% of records, Table 1). Then, the websites “Le Monde des insectes” and “Spipoll” represented, respectively, 17.1% and 12.8% of records.

**Table 1:** Origin of records (*locality* × *year* combinations, 117 combinations in total)

Data source	Number of records	Proportion
“Apoidea‐Gallica” forum	33	28.2
Authors’ naturalist network	23	19.7
“Le Monde des insectes” website	20	17.1
“Spipoll” website	15	12.8
Other websites	11	9.4
Authors’ personal records	10	8.5
Published records	3	2.6
“Réserves Naturelles de France” network	2	1.7

**Figure 2:**
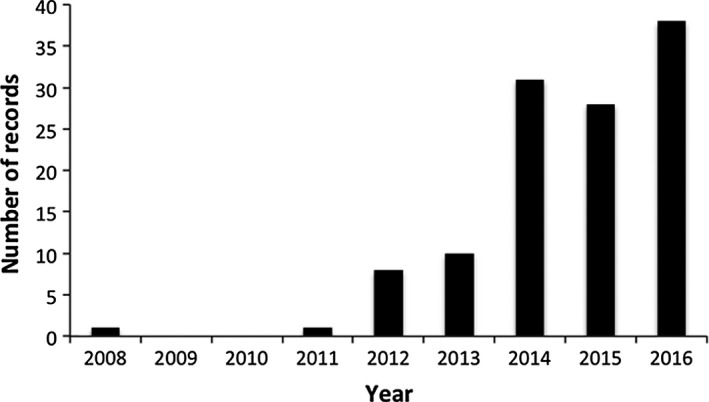
Number of *Megachile sculpturalis* records (i.e., number of localities where observations occurred) per year between 2008 and 2016

**Figure 3:**
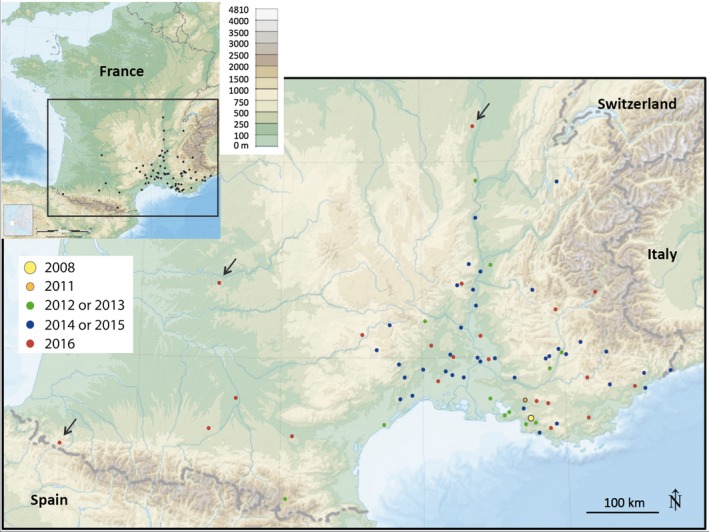
*Megachile sculpturalis* records in France between 2008 and 2016 (status: 31 October 2016). Colors indicate the year when first record occurred in each city (see Table 2). The black arrows show the most distant records from the first record in 2008. Scale bar indicates elevation. See Westrich et al. (2015) for a map of the distribution in Europe

After the first record in 2008 in Allauch (43°20′13″N, 5°28′58″E), the following observation was made in 2011 in Aix‐en‐Provence (43°31′52″N, 5°27′14″E) (Figures 2 and 3). The 2014–2016 period gathers most of records (97 records, i.e., 86.3% of all records, Figure 2). Observations were made between the 19th of June and the 10th of September but mostly occurred in July (48.7%) and August (27.4%).

Overall, *M. sculpturalis* was recorded in 72 French cities (Figure 3 and Table 2). In some cities, the species was recorded at several localities. The total number of localities was 83 (Table 2). The most northern record was located 335 km from Allauch, in Mâcon (46°18′22″N, 4°49′53″E) (one individual in 2016). The most western record was 520 km from Allauch, in Alçay‐Alçabéhéty‐Sunharette (43°05′46″N, 0°54′27″O), western Pyrenees (one individual in 2016). The altitude of the localities ranged from a few meters above sea level (e.g., in Agde, 43°18′39″N, 3°28′33″E) to 1,540 m (one individual found in 2013 in Matemale, 42°35′16″N, 2°07′10″E, eastern Pyrenees).

**Table 2:** French cities with at least one *Megachile sculpturalis* record between 2008 and 2016

City name	Years when observations occurred	Number of localities
Agde	2012	1
Aiguines	2016	1
Aix‐en‐Provence	From 2011 to 2016	4
Alçay‐Alçabéhéty‐Sunharette	2016	1
Allan	2014	1
Allauch	2008, 2012, 2014	1
Allons	2015	1
Antibes	2015	1
Arzens	2016	1
Aubagne	2013	1
Avignon	From 2014 to 2016	4
Barcillonnette	2016	1
Beaucaire	2014	1
Boffres	2015	1
Bouc‐Bel‐air	2014	1
Bouillargues	2014, 2015	1
Boulc	2015	1
Cazilhac	2015, 2016	1
Chabeuil	2013	1
Châteauneuf‐les‐Martigues	2013, 2015	1
Cournonterral	2015, 2016	1
Cruas	2015, 2016	1
Daglan	2016	1
Digne‐les‐Bains	2014, 2016	1
Embrun	2016	1
Etoile	2014, 2015	1
Florac	2014	1
Forcalquier	2014	1
Fousseret	2016	1
Gonfaron	2016	1
Istres	From 2013 to 2015	1
Jonquerettes	2016	1
La Ciotat	2014, 2016	1
Lauris	2014	1
Le Castellet	2016	1
Les Mées	From 2012 to 2014	1
Lyon	2013	1
Mâcon	2016	1
Malbosc	2012	1
Mane	2014	1
Manosque	2013	1
Marignane	From 2012 to 2016	1
Marseille	2012, 2015, 2016	3
Mas‐de‐Londres	2014	1
Matemale	2013	1
Méjannes‐lès‐Alès	2016	1
Menton	2015	1
Montcel	2015	1
Montpellier	From 2014 to 2016	3
Mostuéjouls	2016	1
Mouans‐Sartoux	2016	1
Nîmes	2014, 2015	2
Peyruis	2014	1
Pourrières	2016	1
Privas	2016	1
Puimichel	2014	1
Roussillon	2015, 2016	1
Saint‐Antonin‐sur‐Bayon	2016	1
Saint‐Julien‐de‐Peyrolas	2015	1
Saint‐Maximin	2016	1
Saint‐Priest	2014	1
Sardan	2015, 2016	1
Seillans	2014	1
Sérignan‐du‐Comtat	2016	1
Signes	2012, 2014, 2015, 2016	1
Toulouse	2016	1
Trèves	2015	1
Uzès	2015	1
Velleron	2015, 2016	1
Vence	2014	1
Vergèze	2016	1
Villeneuve‐lès‐Avignon	2014	1

The table gives the year(s) when observation(s) occurred and the number of localities where *Megachile sculpturalis* was recorded.

A total of 59 feeding events (*locality* × *year* × *visited plant species* combinations) were recorded. Twenty plant species belonging to eight families were visited for nectar and/or pollen (Table 3). Overall, 14 taxa were native and six were introduced species from Asia for ornamental purposes. The most visited plants belonged to the genus *Lavandula* (22 records, i.e., 37.3% of plant records). *Sophora japonica* was mentioned 10 times (16.9% of plant records). The six visits to species from the genus *Scabiosa* represented 10.2% of plant records. Two studies reported analyses of pollen samples collected in France either in brood cells or directly from the abdomen of a specimen (Andrieu‐Ponel, et al., 2016; Westrich et al., 2015). Both studies suggested that the larval pollen provision contained a majority of pollen from *S. japonica*.

**Table 3:** Number of feeding events (*locality × year × visited plant species* combinations) and origin of each taxon visited by *Megachile sculpturalis* in France

Family	Species (or genus)	Number of records	Origin	Female/male
Asteraceae	*Centaurea* L.	1	Native	M
	*Cirsium arvense* (L.) Scop.	1	Native	M
	*Cirsium eriophorum* (L.) Scop.	1	Native	F
	*Serratula tinctoria* L.	1	Native	M
Caprifoliaceae	*Cephalaria leucantha* (L.) Schrad. ex Roem. & Schult.	1	Native	M
	*Scabiosa atropurpurea* L.	2	Native	F, M
	*Scabiosa columbaria* L.	2	Native	M
	*Scabiosa* L.	2	Native	M
Fabaceae	*Sophora japonica* L.	10	Introduced from Asia	F
	*Wisteria sinensis* (Sims) Sweet	1	Introduced from Asia	F, M
Lamiaceae	*Clinopodium acinos* (L.) Kuntze	1	Native	M
	*Lavandula* L.	22	Native	F, M
	*Origanum vulgare* L.	1	Native	M
	*Perovskia* Kar.	4	Introduced from Asia	F, M
	*Salvia* L.	1	Native	M
	*Vitex agnus‐castus* L.	2	Native	F, M
Malvaceae	*Firmiana simplex* (L.) W.Wight	1	Introduced from Asia	F
Rosaceae	*Rubus* L.	1	Native	M
Sapindaceae	*Koelreuteria paniculata* Laxm.	1	Introduced from Asia	F, M
Scrophulariaceae	*Buddleja davidii* Franch.	3	Introduced from Asia	M

In our survey, a total of 59 records provided information on visited plants at the species or genus level. The last column indicates the gender of the individuals observed on the flowers. Males visit flowers to drink nectar. Females visit flower to drink and collect nectar and/or to collect pollen.

**Figure 4:**
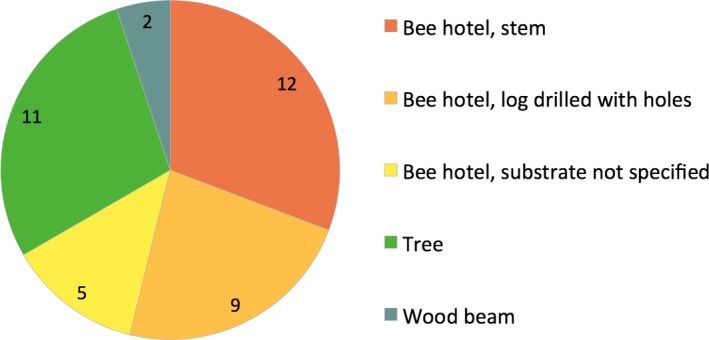
Distribution of nesting events (*locality × substrate* combinations, *N* = 39 in total) according to the substrate used

We compiled 39 nesting events (*locality* × *substrate* combinations) in 35 different localities. In 26 cases, nesting occurred in a human‐made nesting structure (“bee hotels”) (Figure 4). Stems were used in 12 cases (*Arundo* sp. were used four times and *Sambucus* sp. were used twice). Logs drilled with holes were used in nine cases (*Quercus suber* and *Pinus* sp. were both recorded once). For the remaining “bee hotel” cases, we did not have information about the type of cavity used. In 11 cases, nests were located in an old tree. The species *Quercus pubescens* and *Quercus ilex* were both reported twice, and we did not have information about the tree species used for the other events. In two cases, nesting occurred in a wooden beam.

Events of nest occupation or eviction of native wild bees from their nests were recorded in four localities (two times for both *Osmia* sp. and *Xylocopa* sp.).

## 4 DISCUSSION

Since its first record in 2008 (Vereecken & Barbier, 2009), the geographical range of *M. sculpturalis* has extended remarkably, now occupying a third of continental France. In addition, *M. sculpturalis* is now present in Austria, Italy, Germany, and Switzerland and is therefore well established in mainland Europe. Several bee species have been introduced, accidentally or deliberately, outside their native range due to human activities, mainly in North and South America and in Oceania (Goulson, 2003; Russo, 2016). In the northeastern USA, for example, Bartomeus et al. (2013) identified 20 bee species not native to the USA. To our knowledge, *M. sculpturalis* is the first introduced bee species in France and, more globally, in Europe.

Our study illustrates the power of citizen science, defined as the involvement of volunteers in research (Dickinson, Zuckerberg, & Bonter, 2010), to assess the ongoing range expansion of *M. sculpturalis*. Citizen science offers several advantages to collect data on species distribution, such as an extension of spatial and temporal sampling effort (including data collection on private lands such as gardens) and time and cost reduction (Dickinson et al., 2010). Citizen‐collected data can make a major contribution to understand changes in species’ distributions and, more specifically, biological invasions (see Kamenova et al., 2017 for a general review, Roy et al., 2015 for a review regarding Great Britain, and Ashcroft, Gollan, & Batley, 2012 for the study on bee species recently introduced to Australia). In our study, the data we collected fall into the “opportunistic data” category, that is, data collected without standardized protocol and sampling effort. Interest of such datasets is often limited by the lack of “absence data” (e.g., Sequeira, Roetman, Daniels, Baker, & Bradshaw, 2014). Although some entomologists contacted us to report the nonobservation of *M. sculpturalis* in their prospecting area (these “absence data” came from eastern France [Côte d'Or and Jura], from central France [Loir‐et‐Cher and Paris], and from the West [Bretagne and Pays de la Loire]), no active search with standardized protocol has been made in the whole country and we did not have a complete “absence dataset” at the national scale. However, the network of entomologists we communicated with during this study covered the whole France and this contributed to the quality and the reliability of our dataset and of the final map we obtained.

The velocity of the spread of *M. sculpturalis* is not unprecedented for a wild bee species. Indeed, previously reported cases, notably *B. terrestris* in South America, also spread fast (about 100 km per year, Geslin & Morales, 2015). This important dispersal ability is linked to its life history traits. *M. sculpturalis* is a large bee species, polylectic, and it nests in stems or in cavities in wood. As suggested by Quaranta et al. (2014), this latter trait probably favored the transportation of this species through infested trunk by ships, train, or road. This suggestion also fits our data as its spread northward closely matches the Rhone valley, one of the biggest French river, and a major waterway.

Although qualified as polylectic in the literature, palynologic analyses of the larval provision retrieved from literature indicated a preference for *S. japonica* (Andrieu‐Ponel et al., 2016; Westrich et al., 2015). This species is native to China and Korea (Orwa, Mutua, Kindt, Jamnadass, & Simons, 2009) and planted in Europe as an ornamental tree. It flourishes in France in summer, at the same time as the flight period of *M. sculpturalis*. The tendency for introduced bees to forage preferentially on introduced plant species has been reported in several cases (e.g., Stout et al., 2002). The planting of *S. japonica* individuals as ornamental trees could promote the spread of *M. sculpturalis*, and stakeholders may favor native plants and trees in green spaces. Reported cases of biological invasions have already shown to what extent mutualistic interactions and pollination networks could be disrupted by introduced bees (Aizen et al., 2014; Cane & Tepedino, 2017; Geslin et al., 2017; Traveset & Richardson, 2014). The promotion of the reproduction of exotic plants such as *S. japonica* by the spread of *M. sculpturalis* should thus be monitored (see also Quaranta et al., 2014).

*Megachile sculpturalis* is known to develop aggressive behavior toward other species and compete for their nesting sites (Laport & Minckley, 2012; Roulston & Malfi, 2012). We also report several events of nest occupation or eviction of *Osmia* sp. and *Xylocopa* sp. individuals by *M. sculpturalis*. Few concerns are generally paid to bee invasions due to their crucial role as pollinators. But our study shows that competition for nesting sites between *M. sculpturalis* and native bee species could occur. In France, species such as *Xylocopa* spp., *Lithurgus* spp., *Osmia* spp., *Megachile lagopoda*, and some *Anthidium* spp. could be negatively affected. Moreover, introduced bees may also spread diseases to native bees (e.g., Graystock, Blane, McFrederick, Goulson, & Hughes, 2015; Singh et al., 2010).

Taken together, *M. sculpturalis* presence and spread may have deleterious consequences for native bees and its progression should be carefully monitored. Its high detectability (large species occurring in many habitats, including urban areas) and its easy identification make this species particularly appealing and appropriate for citizen science. We suggest continuing and amplifying the monitoring of the species. In parks and gardens, the visual inspection of *S. japonica* flowers and bee hotels could provide an effective means of detecting the species in an area but prospecting in natural areas (e.g., forests) should not be neglected in order to obtain the most accurate picture of the species’ spread and its impacts.

## CONFLICT OF INTEREST

None declared.

## AUTHOR CONTRIBUTIONS

Violette Le Féon and Benoît Geslin designed the study and wrote the first draft of the manuscript. Violette Le Féon, Matthieu Aubert, David Genoud, Valérie Andrieu‐Ponel, Paul Westrich, and Benoît Geslin provided their own observations on *Megachile sculpturalis* and collated data from other observers. All authors contributed substantially to this manuscript.

## DATA ACCESSIBILITY

*Megachile sculpturalis* records reported in this study are available from the Dryad Digital Repository: https://doi.org/10.5061/dryad.7bq1q.
